# Association of prolonged ECMO bridging and perioperative factors with dysphagia and survival after lung transplantation

**DOI:** 10.1371/journal.pone.0353273

**Published:** 2026-07-09

**Authors:** Woo Chul Son, Garam Hong, Sang-Bum Hong, Cheon Ji Kang, Ho Cheol Kim, Won Kim

**Affiliations:** 1 Department of Rehabilitation Medicine, Asan Medical Center, University of Ulsan College of Medicine, Seoul, Republic of Korea; 2 Department of Pulmonary and Critical Care Medicine, Asan Medical Center, University of Ulsan College of Medicine, Seoul, Republic of Korea; 3 Department of Rehabilitation Medicine, Daejeon Rehabilitation Hospital, Daejeon, Republic of Korea; The University of Kansas Health Systems St. Francis Campus, UNITED STATES OF AMERICA

## Abstract

**Objectives:**

To evaluate the association between perioperative variables, including extracorporeal membrane oxygenation bridge-to-transplantation (ECMO BTT), and the development of dysphagia after lung transplantation (LT), and to examine the association between postoperative dysphagia and long-term survival.

**Methods:**

This retrospective, single-center study included adult patients who underwent LT between November 2008 and December 2022. Swallowing function was assessed using the Functional Oral Intake Scale (FOIS) on postoperative day (POD) 28, POD 60, and at hospital discharge. Patients were classified into dysphagia (FOIS ≤3) and non-dysphagia (FOIS >3) groups based on FOIS at POD 28. Perioperative variables associated with dysphagia were examined using multivariable logistic regression. Survival outcomes were assessed using Kaplan–Meier analysis.

**Results:**

Among the 124 included patients, 35.9% had dysphagia at POD 28, whereas 87.1% achieved functional oral intake by hospital discharge. Prolonged ECMO BTT (≥14 days), tracheostomy before POD 28, female sex, and postoperative pneumonia were independently associated with dysphagia at POD 28. Patients with dysphagia at POD 28 showed significantly poorer unadjusted 1-year and 2-year survival than those without dysphagia.

**Conclusions:**

Dysphagia at POD 28 after LT was associated with prolonged ECMO BTT, tracheostomy before POD 28, postoperative pneumonia, and poorer unadjusted survival. FOIS at POD 28 may serve as a clinically meaningful marker of delayed recovery and increased clinical vulnerability.

## Introduction

Lung transplantation (LT) has become an established treatment for a range of end-stage lung diseases, with survival rates steadily improving as a result of advances in surgical techniques and perioperative management [1,2]. In parallel, the expansion of transplant indications and refinement of bridging strategies have enabled an increasing number of patients to undergo transplantation [2]. As post-transplant survival has improved and the burden of long-term complications has grown, attention has been directed toward long-term outcomes and quality of life (QOL) in LT recipients. Accordingly, efforts to identify post-transplant complications and associated risk factors that may adversely affect these outcomes have intensified [3–5].

A broad spectrum of complications may occur after LT, including vascular, respiratory, cardiovascular, and infectious events, with infection remaining a leading cause of post-transplant mortality [4,5]. Dysphagia represents another clinically important complication and has been reported to occur with relatively high frequency after thoracic surgeries, including LT [6,7]. Dysphagia-related aspiration may contribute not only to infectious complications such as pneumonia but also to the development and progression of chronic lung allograft dysfunction, thereby adversely affecting both short- and long-term outcomes [5,7].

Given its clinical relevance, recent studies have increasingly sought to clarify the association between perioperative factors and the development of dysphagia [6]. However, the relationship between patient characteristics, perioperative management, and dysphagia in LT recipients has not been fully elucidated. In particular, the impact of specific perioperative characteristics, such as extracorporeal membrane oxygenation bridge-to-transplantation (ECMO BTT), on the risk of dysphagia remains incompletely characterized [8,9].

Therefore, the present study aimed to evaluate the association between perioperative variables, including ECMO BTT, and the development of dysphagia in lung transplant recipients, as well as to examine the relationship between clinically identified dysphagia and long-term survival.

## Methods

### Study design and baseline characteristics

This retrospective, single-center study included adult patients who underwent LT at a tertiary referral hospital between November 2008 and December 2022. The study protocol was approved by the institutional review board (IRB No. 2024-0192), and the requirement for informed consent was waived due to the retrospective design. Data were accessed for research purposes on 22 February 2024, and the authors did not have access to information that could identify individual participants during or after data collection. All research procedures were conducted in accordance with the Declaration of Helsinki and complied with the Strengthening the Reporting of Observational Studies in Epidemiology (STROBE) guidelines.

All eligible patients were identified through transplant records and screened for inclusion. Eligibility criteria were age ≥ 18 years and receipt of LT. Patients were excluded if they had (1) central nervous system lesions that could potentially impact swallowing function or (2) died before postoperative day (POD) 28, precluding adequate assessment of postoperative swallowing status.

Demographic variables, including age, sex, underlying disease leading to LT, smoking history, and type of transplantation (lung-only or combined transplantation), were obtained through retrospective review of electronic medical records. Clinical variables included the presence of tracheostomy, postoperative intensive care unit (ICU) length of stay, total hospital length of stay, postoperative hospital days, perioperative ventilator days, occurrence of postoperative pneumonia within 28 days, Acute Physiology and Chronic Health Evaluation II (APACHE II) score at ICU admission, extracorporeal membrane oxygenation bridge-to-transplantation (ECMO BTT), and survival status at 1 and 2 years after transplantation.

## Evaluation of postoperative swallowing function

Postoperative swallowing function was assessed using the Functional Oral Intake Scale (FOIS) at POD 28, POD 60, and before hospital discharge. Additional swallowing-related variables included the presence of vocal cord palsy and whether a videofluoroscopic swallowing study (VFSS) was performed. The FOIS is a validated and reliable ordinal scale developed in 2005 to evaluate functional oral intake and has been widely used in studies of dysphagia after solid organ transplantation [10,11]. FOIS level 1 indicates no oral intake with complete dependence on tube feeding; level 2 allows minimal or therapeutic oral intake with tube feeding; level 3 denotes consistent oral intake that remains nutritionally insufficient and requires supplemental tube feeding; level 4 represents total oral intake without tube feeding but restricted to a single food consistency; level 5 indicates total oral intake of multiple consistencies with dietary modification or compensatory strategies; level 6 reflects total oral intake of multiple consistencies without special preparation but with avoidance of specific foods; and level 7 corresponds to unrestricted total oral intake.

For analytical purposes, FOIS levels 1–3 were classified as the dysphagia group, and levels 4–7 as the non-dysphagia group. This categorization reflects the ability to achieve functional oral intake independent of tube feeding and is consistent with prior studies evaluating dysphagia in critically ill populations [11].

After LT, patients progressed to oral feeding according to the institution’s standard postoperative care protocol. In the absence of contraindications to oral intake, such as ileus or medical instability, oral feeding was gradually advanced, provided that no clinical signs of aspiration were observed. When features suggestive of dysphagia were identified, VFSS was performed, and swallowing rehabilitation was initiated based on examination findings.

## Statistical analysis

Continuous variables are presented as medians with interquartile ranges (IQRs), and categorical variables as frequencies and percentages. The normality of continuous variables was assessed prior to analysis. Based on FOIS scores at POD 28, patients were categorized into dysphagia and non-dysphagia groups, and baseline characteristics were compared accordingly. Categorical variables were analyzed using the chi-square test or Fisher’s exact test, as appropriate. Continuous variables were compared using the independent *t*-test or Mann–Whitney *U* test.

To identify factors associated with dysphagia, univariable logistic regression analyses were initially performed for all candidate variables. Variables with a P-value of ≤0.05 in univariable analyses were entered into multivariable logistic regression models. To address potential multicollinearity related to ECMO exposure, two separate multivariable models were constructed: one including ECMO BTT status and another incorporating prolonged BTT, defined as ECMO support for ≥14 days.

Survival outcomes were analyzed using Kaplan–Meier methods, with comparisons between dysphagia and non-dysphagia groups. All statistical analyses were conducted using SPSS version 21 (IBM Corp., Armonk, NY, USA), and a two-sided P-value of <0.05 was considered statistically significant.

## Results

**Fig 1:**
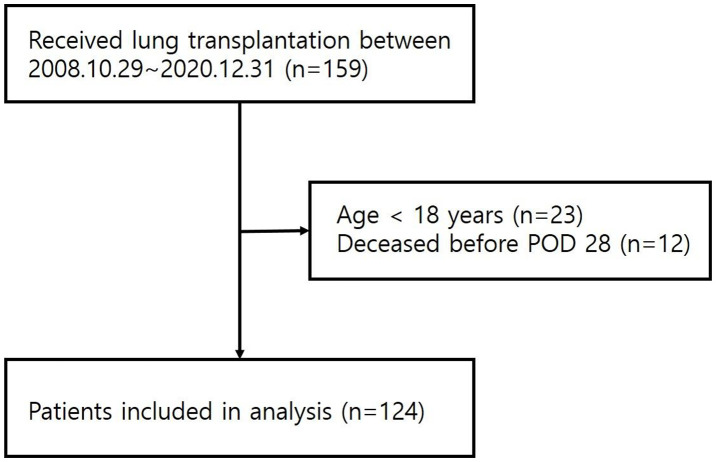
Flow Diagram of Patient Inclusion.

During the study period, 159 patients underwent LT. Of these, 35 were excluded, including 23 aged <18 years and 12 who died before POD 28. The final analysis therefore included 124 patients (Fig 1). Baseline characteristics of the study population are summarized in Table 1. The median age was 55 years (IQR, 43–62), and 111 patients (89.5%) underwent lung-only transplantation. At POD 28, 80 patients (64.5%) were classified into the non-dysphagia group (FOIS >3). By the time of hospital discharge, the proportion of patients achieving functional oral intake had increased to 108 (87.1%).

**Table 1:** Baseline characteristics.

Variables	Median (IQR) or n (%) (n = 124)
Age at operation, years	55 (43, 62)
Female sex	41 (33.1)
Type of transplantation	
Lung only	111 (89.5)
Combined transplantation	13 (10.5)
Diagnosis	
Interstitial lung disease	80 (64.5)
Bronchiolitis obliterans syndrome	9 (7.3)
Pulmonary vascular disease	9 (7.3)
Others	26 (21.0)
Smoking history (pack years)	2.3 (0, 30)
Total hospital days	77 (53, 123)
Postoperative hospital stay, days	60 (37, 99)
Postoperative ICU stay, days	18 (11, 28)
Perioperative ventilator use, days	19 (11, 39)
Perioperative BTT insertion	69 (55.6)
BTT insertion ≥ 14 days	32
BTT insertion < 14 days	37
Tracheostomy	72 (58.1)
Postoperative pneumonia	70 (56.5)
Vocal cord palsy	15 (12.1)
APACHE score at ICU admission*	20 (16, 28)
FOIS>3 at POD 28	80 (64.5)
FOIS>3 at POD 60	100 (80.6)
FOIS>3 at discharge	108 (87.1)
Performed postoperative VFSS	40 (32.3)

*31 APACHE score missing.

ICU, Intensive care unit; BTT, bridge-to-transplantation; APACHE-II, Acute Physiology and Chronic Health Evaluation-II; FOIS, functional oral intake scale; POD, postoperative day; VFSS, videofluoroscopic swallowing study.

Table 2 compares the clinical characteristics of patients with dysphagia (FOIS ≤3) and those without dysphagia (FOIS >3) at POD 28. The type of transplantation and female sex differed significantly between groups. Patients in the dysphagia group had significantly longer total hospital days, postoperative hospital days, postoperative ICU days, and perioperative ventilator days. In addition, the proportion of patients who underwent ECMO BTT and the incidence of postoperative pneumonia within 28 days were significantly higher in the dysphagia group.

**Table 2:** Characteristics according to FOIS at POD 28.

Variables	Median (IQR) or n (%)		P
	FOIS ≤3 (dysphagia)	FOIS >3 (non-dysphagia)	
	(n = 44)	(n = 80)	
Age at operation	57 (45, 63)	55 (43, 62)	0.45
Female sex	21 (47.7)	20 (25.0)	0.02*
Type of transplantation			0.01*
Lung only	35 (79.5)	76 (95.0)	
Combined transplantation	9 (20.5)	4 (5.0)	
Diagnosis			
Interstitial lung disease	27 (61.4)	53 (66.3)	
Bronchiolitis obliterans syndrome	4 (9.1)	5 (6.3)	
Pulmonary vascular disease	4 (9.1)	5 (6.3)	
Other	16 (36.4)	10 (12.5)	
Smoking history	0 (0, 18)	5 (0, 30)	0.26
Total hospital stay	128 (90, 195)	63 (40, 90)	<0.01*
Postoperative hospital stay	103 (70, 156)	46 (34, 69)	<0.01*
Postoperative ICU stay	32 (21, 47)	13 (9, 21)	<0.01*
Perioperative ventilator use	35 (15, 69)	15 (8, 29)	<0.01*
BTT (vs. non-BTT)	33 (75.0)	36 (45.0)	<0.01*
BTT			
None	11	44	
< 14 days	11	21	
≥ 14 days	22	15	
Tracheostomy	39 (88.6)	33 (41.3)	<0.01*
Postoperative pneumonia	36 (81.8)	34 (42.5)	<0.01*
Vocal cord palsy	4 (9.1)	11 (13.8)	0.57
APACHE score at ICU admission **	21 (19.3, 28.8)	19 (15, 25.8)	0.16
Performed postoperative VFSS	27 (61.4)	13 (16.3)	<0.01*

*p-value <0.05.

**14 missing for dysphagia group, 17 missing for the non-dysphagia group.

For categorical variables, the χ² test or Fisher’s exact test was used, and for continuous variables, the independent *t*-test or Mann–Whitney *U* test was applied as appropriate.

ICU, intensive care unit; BTT, bridge-to-transplantation; APACHE-II, Acute Physiology and Chronic Health Evaluation-II; FOIS, functional oral intake scale; POD, postoperative day; VFSS, videofluoroscopic swallowing study.

The results of the logistic regression analyses evaluating factors associated with dysphagia at POD 28 are presented in Table 3. In univariable analyses, type of transplantation, female sex, ECMO BTT, prolonged ECMO BTT (>14 days), tracheostomy before POD 28, and postoperative pneumonia within 28 days were significantly associated with dysphagia (all P < 0.05). Due to multicollinearity between ECMO BTT and prolonged ECMO BTT, these variables were not included simultaneously in multivariable models.

**Table 3:** Multivariable logistic regression analyses for FOIS ≤ 3 at POD 28.

	Univariable			Multivariable-1			Multivariable-2		
Variables	OR	95% CI	P	OR	95% CI	P	OR	95% CI	P
Age at operation	0.989	0.960–1.018	0.45						
Smoking history	1.013	0.991–1.036	0.26						
Combined transplantation	4.886	1.408–16.951	0.01*	3.712	0.873–15.789	0.08*	3.039	0.708–13.039	0.14
Female sex	2.739	1.258–5.966	0.01*	2.811	1.106–7.146	0.03*	3.108	1.222–7.906	0.02*
BTT (vs. non-BTT)	3.667	1.628–8.260	<0.01*	2.170	0.858–5.488	0.10			
Perioperative BTT insertion									
None									
< 14 days	0.879	0.379–2.037	0.76						
≥ 14 days	4.333	1.918–9.791	<0.01*				3.037	1.18–7.81	0.02*
Tracheostomy (<POD 28)	11.109	3.958–31.178	<0.01*	2.931	1.152–7.456	0.02*	2.933	1.146–7.510	0.03*
Postoperative pneumonia (<POD 28)	4.482	2.103–11.148	<0.01*	4.293	1.657–11.124	<0.01*	4.681	1.760–12.447	<0.01*
Vocal cord palsy	1.594	0.476–5.340	0.45						
APACHE-II score	0.960	0.907–1.017	0.16						

*p-value <0.05, logistic regression analysis.

Multivariable-1: including BTT (vs. non-BTT), multivariable-2: including BTT ≥ 14 days.

CI, confidence interval; BTT, bridge-to-transplantation; APACHE-II, Acute Physiology and Chronic Health Evaluation-II; FOIS, functional oral intake scale; POD, postoperative day.

In the multivariable model including ECMO BTT (Model 1), female sex, tracheostomy before POD 28, and postoperative pneumonia within 28 days were independently associated with dysphagia at POD 28. In the multivariable model including prolonged ECMO BTT (>14 days) (Model 2), female sex, prolonged ECMO BTT, tracheostomy before POD 28, and postoperative pneumonia within 28 days remained independently associated with dysphagia.

**Fig 2:**
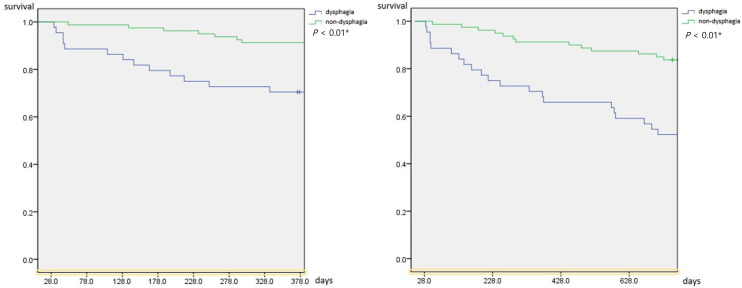
One- and Two-Year Survival According to Dysphagia Status at POD 28. Kaplan–Meier survival curves comparing dysphagia (FOIS ≤3) and non-dysphagia (FOIS >3) groups at POD 28. The left panel shows 1-year survival, and the right panel shows 2-year survival. Both analyses demonstrated significantly lower survival in the dysphagia group (p < 0.01, log-rank test).

Kaplan–Meier survival analyses demonstrated significantly lower 1-year and 2-year survival rates among patients with dysphagia at POD 28 compared with those without dysphagia (Fig 2).

## Discussion

This study evaluated the association between perioperative variables and the development of dysphagia in LT recipients and examined the impact of clinically identified dysphagia at POD 28 on survival outcomes. Although previous studies have reported associations between dysphagia and selected perioperative characteristics in LT recipients, data remain limited in high-risk populations, particularly patients undergoing ECMO BTT, and in relation to subsequent clinical course and survival outcomes [6,8,9]. In the present study, most patients with dysphagia at POD 28 were able to resume oral feeding by hospital discharge. Nevertheless, prolonged ECMO BTT and postoperative pneumonia were significantly associated with dysphagia, and dysphagia defined by FOIS at POD 28 was associated with poorer 1- and 2-year survival. These findings underscore the importance of early dysphagia surveillance and timely intervention in vulnerable, critically ill LT recipients, particularly those requiring prolonged ECMO support or experiencing postoperative pneumonia. Moreover, the observed association between POD 28 FOIS and long-term outcomes suggests that FOIS at this time point may serve as a clinically accessible indicator of recovery trajectory after LT.

Assessment of FOIS at POD 28, POD 60, and at discharge provides clinically meaningful insight into the timing and progression of oral feeding recovery after LT. Although 35.9% of patients exhibited clinically significant dysphagia at POD 28, swallowing function improved gradually, with approximately two-thirds of these patients achieving functional oral intake by discharge. This pattern suggests that early post-transplant dysphagia more often reflects transient functional impairment rather than irreversible surgical complications. Previous studies have reported vocal cord palsy rates of approximately 9% after LT and recurrent laryngeal nerve injury rates of less than 3% after heart transplantation, with most cases demonstrating spontaneous recovery [12,13]. Collectively, these findings support the notion that post-LT dysphagia is more commonly attributable to multifactorial mechanisms—such as ICU-acquired weakness, prolonged intubation, and global deconditioning—rather than direct surgical nerve injury [3,6,7,14]. Importantly, even when transient dysphagia is present at POD 28, appropriate postoperative management and prevention of secondary complications, including pneumonia, may facilitate recovery of oral intake by discharge.

Postoperative pneumonia occurred significantly more frequently in patients with dysphagia at POD 28 (Table 2). The pathogenesis of pneumonia after LT is multifactorial and includes ischemia–reperfusion injury, immunosuppression, prolonged mechanical ventilation, and oropharyngeal dysphagia [15]. In cardiac and thoracic surgical populations, postoperative pneumonia is widely recognized as a surrogate marker of dysphagia [16,17]. Consistent with prior literature, our findings suggest that the occurrence of pneumonia in LT recipients should prompt targeted assessment of oropharyngeal function to optimize clinical management. A higher proportion of female patients was also observed in the dysphagia group. This finding may be influenced by the distribution of underlying conditions, as women accounted for 55.6% of patients with bronchiolitis obliterans and 46.2% of those undergoing combined transplantation. Bronchiolitis obliterans, commonly occurring after bone marrow transplantation, has been associated with poorer post-LT outcomes, while combined heart–lung transplantation, although associated with survival outcomes comparable to lung-only transplantation, is often characterized by worse preoperative functional status and greater deconditioning [18,19]. These factors may have contributed to poorer swallowing function at 4 weeks postoperatively.

The relatively high prevalence of ECMO BTT in our cohort should be interpreted within the context of the Korean lung allocation system, which has historically prioritized medical urgency. Under this system, candidates requiring mechanical ventilation and/or ECMO have comprised a relatively large proportion of transplant recipients in Korea, and this national allocation context may partly explain the high proportion of ECMO-bridged patients in our study [20]. Against this background, patients requiring ECMO BTT are generally recognized to have lower pretransplant functional reserve than non-BTT recipients [21,22]. In our cohort, ECMO BTT was also more common in the dysphagia group (Table 2). However, multivariable analyses demonstrated that prolonged ECMO BTT (>14 days), rather than ECMO BTT itself, was independently associated with dysphagia (Table 3). This finding is consistent with previous reports suggesting favorable outcomes when ECMO BTT is limited to ≤14 days, as well as studies showing that prolonged ECMO support, rather than ECMO use per se, increases the risk of dysphagia and aspiration in critically ill populations, including patients with COVID-19 [22,23]. Taken together, these findings suggest that prolonged ECMO BTT may reflect both the burden of extended extracorporeal support and greater underlying illness severity, thereby underscoring the need for early and systematic assessment of oropharyngeal function in this high-risk subgroup [24].

When stratified by dysphagia status at POD 28, patients with dysphagia exhibited significantly poorer unadjusted 1- and 2-year survival compared with those without dysphagia. Dysphagia likely reflects not only local oropharyngeal dysfunction but also the cumulative impact of prolonged critical illness, including ICU-acquired weakness, extended intubation, and systemic frailty [15]. Accordingly, persistent dysphagia at POD 28 may be interpreted as a surrogate marker of delayed functional recovery and increased overall disease severity rather than an isolated swallowing disorder. This interpretation aligns with previous studies demonstrating an association between dysphagia and long-term survival after LT [6,7]. Additionally, when inadequately assessed or managed, dysphagia may directly contribute to adverse outcomes through malnutrition and pulmonary complications. In this context, FOIS assessment at POD 28—an easily applicable functional outcome measure—may be valuable for early identification of high-risk patients, and early implementation of specialized swallowing evaluation and rehabilitation may represent an important strategy for improving long-term outcomes after LT. However, the association between POD 28 dysphagia and post-transplant survival should be interpreted with caution, as prolonged ECMO BTT may have contributed both to the development of dysphagia and to poorer post-transplant survival [25].

## Limitations

This study has several limitations. First, the retrospective design limited the ability to capture all factors that may have influenced FOIS scores or mortality. Clinically relevant variables, such as gastroesophageal reflux disease and bronchiolitis obliterans—both recognized risk factors for allograft dysfunction after LT—were not included in the analysis. Future prospective studies incorporating these variables may provide a more comprehensive understanding of dysphagia-related outcomes. In addition, this single-center cohort included a high proportion of patients who underwent ECMO BTT, indicating a relatively high-acuity transplant population. Because ECMO BTT itself may reflect greater underlying disease severity, it could have acted as an important confounder in the observed associations between dysphagia and clinical outcomes. Therefore, the generalizability of our findings to broader lung transplant populations or to centers with different bridging practices may be limited. Second, FOIS does not fully characterize swallowing physiology, as it cannot detect silent aspiration or penetration. Although FOIS is readily applicable in routine clinical practice, it lacks the objective visualization provided by VFSS or FEES, and instrumental swallowing assessments were performed in only a subset of patients in this study. Nevertheless, FOIS has been shown to serve as a meaningful global indicator of swallowing function in prior studies and was therefore selected as the primary outcome measure in this analysis [10]. Future studies should incorporate systematic instrumental assessments to more precisely characterize swallowing dysfunction. Finally, preoperative swallowing function was not assessed, and baseline differences in oropharyngeal function may have influenced postoperative swallowing outcomes.

## Conclusion

In this retrospective study, most LT recipients with dysphagia at POD 28 recovered swallowing function by POD 60 or by hospital discharge. Prolonged ECMO BTT and postoperative pneumonia were independently associated with dysphagia at POD 28. Dysphagia at POD 28 was also associated with poorer unadjusted 1- and 2-year survival and may serve as a clinical marker of delayed recovery and greater illness severity rather than an isolated swallowing disorder. Future prospective studies with systematic instrumental swallowing assessment and adjusted survival analyses are needed to further clarify these associations.
