# Supplementary material for: Association of prolonged ECMO bridging and perioperative factors with dysphagia and survival after lung transplantation
Source: PLoS One. 2026 Jul 9;21(7):e0353273. doi: 10.1371/journal.pone.0353273 (PMC13349096; doi:10.1371/journal.pone.0353273)
Supplement: Attachment — Submitted filename: Response letter.docx [file pone.0353273.s002.docx]

Journal Requirements: PLOS ONE Editorial Office

We thank the Editor for these important editorial comments and guidance.

We have revised the manuscript to conform to PLOS ONE style and formatting requirements, including the use of the journal’s formatting templates and file naming conventions.

We have revised the manuscript so that the ethics statement appears only in the Methods section and have removed it from all other sections.

3–6. We appreciate the Editor’s guidance regarding the Data Availability Statement. This study involved sensitive clinical information from lung transplant recipients. In Korea, transplantation-related recipient data are managed within a national regulatory framework involving the Korea Network for Organ Sharing (KONOS). In addition, unrestricted public sharing of the dataset would not be consistent with the IRB-approved protocol and with applicable institutional and national regulations governing transplantation data. We have therefore revised the Data Availability Statement to clarify these restrictions and to provide the contact information of a non-author institutional body through which qualified researchers may request access to de-identified data.

Revised Data Availability Statement:

The data underlying the results presented in this study contain potentially identifying and sensitive clinical information from lung transplant recipients. For ethical and legal reasons, these data cannot be shared publicly. In Korea, transplantation-related recipient data are managed within a national regulatory framework involving the Korea Network for Organ Sharing (KONOS) under the Organ Transplant Act. In addition, public release of the study dataset would be inconsistent with the IRB-approved protocol and applicable institutional and national regulations governing transplantation data. De-identified data may be made available to qualified researchers upon reasonable request, subject to review and approval by the Institutional Review Board of Asan Medical Center and, where applicable, the relevant national authority. Requests for data access should be directed to the Institutional Review Board of Asan Medical Center via its official mail at [irb@amc.seoul.kr](mailto:irb@amc.seoul.kr) or by telephone at +82-2-3010-7157. The authors are not the sole point of contact for data access.

We confirm the following Role of Funder statement:

The funders had no role in study design, data collection and analysis, decision to publish, or preparation of the manuscript.

We reviewed the reviewer-suggested reference(s) and incorporated relevant citation(s) into the revised manuscript where appropriate.

Reviewer response:

Reviewer #1: Dysphagia has an important impact following lung transplantation, and I find this paper interesting and well written. I have some concerns that need to be addressed in the discussion:

1. The proportion of patients bridged with ECMO in this cohort (>50%) is substantially higher than typically reported in lung transplant populations. This raises concerns about the cohort's representativeness and limits the generalizability of the findings. Please address this in the limitations section.

We appreciate this important comment and agree with the reviewer. In our cohort, 69 of 124 patients (55.6%) underwent ECMO BTT, indicating that the study population reflects a relatively high-acuity transplant cohort rather than a broadly representative LT population. We have therefore revised the Limitations section to explicitly acknowledge that the high prevalence of ECMO BTT in this single-center cohort may limit the generalizability of our findings to centers with different case mix, bridging practices, or perioperative management strategies.

Limitations

In addition, this single-center cohort included a high proportion of patients who underwent ECMO BTT, indicating a relatively high-acuity transplant population. Because ECMO BTT itself may reflect greater underlying disease severity, it could have acted as an important confounder in the observed associations between dysphagia and clinical outcomes. Therefore, the generalizability of our findings to broader lung transplant populations or to centers with different bridging practices may be limited.

2. Furthermore, the proportion of patients on prolonged ECMO is also high within the ECMO population, which is usually reserved for the sickest patients, thereby increasing the risk of confounding by indication and reducing the ability to adequately distinguish the independent effect of ECMO from underlying disease severity on the development of dysphagia. Some details on the indication for transplant (CTD-ILD, some of which have known GI involvement or idiopathic interstitial pneumonias), as well as the indication for ECMO and prolonged ECMO, will help contextualize this issue.

We sincerely thank the reviewer for this important comment. We agree that prolonged ECMO support may introduce confounding by indication, as it likely reflects both ECMO-related exposure and greater underlying disease severity.

In response, we revised the Discussion to note that the Korean lung allocation system has historically prioritized medical urgency, which may partly explain the high proportion of ECMO-bridged patients in our cohort. We also agree that patients requiring prolonged ECMO BTT likely represent a particularly severe subgroup, making it difficult to fully distinguish the effect of prolonged ECMO itself from that of underlying illness severity on postoperative dysphagia.

We acknowledge that more detailed information regarding transplant indication and the indication for ECMO would further strengthen the interpretation of our findings. However, because these details were not uniformly available in a structured form across the study period, we were unable to add a reliable indication-based subgroup analysis in the current revision.

Discussion

The relatively high prevalence of ECMO BTT in our cohort should be interpreted within the context of the Korean lung allocation system, which has historically prioritized medical urgency. Under this system, candidates requiring mechanical ventilation and/or ECMO have comprised a relatively large proportion of transplant recipients in Korea, and this national allocation context may partly explain the high proportion of ECMO-bridged patients in our study.(20) Against this background, patients requiring ECMO BTT are generally recognized to have lower pretransplant functional reserve than non-BTT recipients.(21,22) In our cohort, ECMO BTT was also more common in the dysphagia group (Table 2). However, multivariable analyses demonstrated that prolonged ECMO BTT (>14 days), rather than ECMO BTT itself, was independently associated with dysphagia (Table 3). This finding is consistent with previous reports suggesting favorable outcomes when ECMO BTT is limited to ≤14 days, as well as studies showing that prolonged ECMO support, rather than ECMO use per se, increases the risk of dysphagia and aspiration in critically ill populations, including patients with COVID-19.(22,23) Taken together, these findings suggest that prolonged ECMO BTT may reflect both the burden of extended extracorporeal support and greater underlying illness severity, thereby identifying patients who may particularly benefit from early and systematic assessment of oropharyngeal function.(24)

3. The interpretation of the association between dysphagia and post-transplant mortality is adequately discussed as more of a surrogate marker for the severity of illness than causality. Please add the prolonged ECMO confounder here, as pre-transplant ECMO, particularly when prolonged, is associated with worse 1- and 2-year survival (Rando et al. Extracorporeal membrane oxygenation as a bridge to lung transplantation: Practice patterns and patient outcomes. J Heart Lung Transplant 2024;43:77–84). In the present cohort, where ECMO use is highly prevalent, it is likely that ECMO both predisposes to the development of dysphagia and independently impacts mortality.

We sincerely thank the reviewer for this important comment. We agree that prolonged ECMO BTT is an important potential confounder in interpreting the association between dysphagia and post-transplant mortality.

In response, we revised the Discussion to clarify that dysphagia at POD 28 should be viewed as a marker of greater illness severity rather than as an independent causal determinant of mortality. We also added the suggested reference by Rando et al. and noted that prolonged pre-transplant ECMO has been associated with worse post-transplant survival. Accordingly, we tempered our interpretation to acknowledge that prolonged ECMO may have contributed both to dysphagia and to poorer survival in our cohort.

Discussion

However, the association between POD 28 dysphagia and post-transplant survival should be interpreted with caution, as prolonged ECMO BTT may have contributed both to the development of dysphagia and to poorer post-transplant survival.

Reviewer 2

MAJOR CONCERNS:

1. Survival is compared between dysphagia and non-dysphagia groups using Kaplan-Meier analysis only, without multivariable Cox regression. Given that the dysphagia group had significantly more ECMO BTT, pneumonia, and longer ICU stays, the unadjusted survival difference may reflect severity of illness rather than dysphagia per se. Adjusted survival analysis is necessary to support the conclusion that dysphagia independently predicts mortality.

We sincerely thank the reviewer for this important comment. We fully agree that Kaplan–Meier analysis alone does not allow postoperative dysphagia to be interpreted as an independent predictor of mortality.

In the present study, the survival analysis was intended as a descriptive comparison of overall survival according to FOIS-defined swallowing status at POD 28, rather than as a formal adjusted prognostic model. In response to the reviewer’s comment, we revised the manuscript to avoid wording that could imply independent prediction or causality. We now describe the survival findings as an unadjusted association and interpret POD 28 dysphagia primarily as a marker of delayed recovery and greater illness severity rather than as an isolated determinant of mortality.

We also strengthened the Discussion below to acknowledge that the observed survival difference may have been influenced by important confounders, including ECMO bridge-to-transplantation, prolonged ECMO support, postoperative pneumonia, and longer ICU stay. In particular, we added that prolonged ECMO BTT may have contributed both to the development of dysphagia and to poorer post-transplant survival. We agree that adjusted time-to-event analysis would be important in future studies, and we have tempered our conclusions accordingly.

Discussion

Taken together, these findings suggest that prolonged ECMO BTT may reflect both the burden of extended extracorporeal support and greater underlying illness severity, thereby underscoring the need for early and systematic assessment of oropharyngeal function in this high-risk subgroup.(24)

However, the association between POD 28 dysphagia and post-transplant survival should be interpreted with caution, as prolonged ECMO BTT may have contributed both to the development of dysphagia and to poorer post-transplant survival.

Limitation

In addition, this single-center cohort included a high proportion of patients who underwent ECMO BTT, indicating a relatively high-acuity transplant population. Because ECMO BTT itself may reflect greater underlying disease severity, it could have acted as an important confounder in the observed associations between dysphagia and clinical outcomes. Therefore, the generalizability of our findings to broader lung transplant populations or to centers with different bridging practices may be limited.

MINOR CONCERNS:

2. VFSS was performed in only 40 patients (32.3%), and 31 VFSS data points are missing. Reliance on FOIS alone without systematic instrumental assessment in all patients may result in underdiagnosis of silent aspiration, particularly in the non-dysphagia group. This limitation should be more prominently discussed.

We thank the reviewer for this important point. We apologize for the ambiguity in the table. The 31 missing values refer to APACHE-II scores, not VFSS. VFSS was performed selectively in patients with clinical suspicion of dysphagia rather than systematically in all patients. We agree, however, that reliance on FOIS without universal instrumental swallowing assessment may have led to underrecognition of silent aspiration, particularly in patients classified as non-dysphagic based on FOIS alone. We have clarified this point in the revised manuscript and emphasized it more explicitly in the Limitations section.

Limitation

Second, FOIS does not fully characterize swallowing physiology, as it cannot detect silent aspiration or penetration. Although FOIS is readily applicable in routine clinical practice, it lacks the objective visualization provided by VFSS or FEES, and instrumental swallowing assessments were performed in only a subset of patients in this study.

Reviewer #3: In this retrospective single center cohort review, the authors provide an analysis of perioperative factors and their association with postoperative dysphagia in lung transplant recipients, as well as the survival rates in patients with postoperative dysphagia.

Chief amongst the problems with this study include the title itself, with impact implying some degree of causality. This dovetails into the principle challenge to the veracity of this paper. Dysphagia itself is likely reflective of the severity of underlying debility and/or frailty, with resultant residual confounding.

Of note, while the data may not appear to be immediately public, it appears that it should be otherwise easily obtainable from the authors and appropriately guarded for privacy reasons.

At face value, the primary takeaway from this paper appears to be that sicker or otherwise more critically ill patients with prolonged durations of such have increased rates of dysphagia with subsequently lower survival, APACHE-II scores aside. To some degree these findings seem self-evident, which may limit the effect that this publication may have.

We sincerely thank the reviewer for this thoughtful and careful assessment. We agree that the term “impact” in the original title may imply a degree of causality that is not fully supported by our retrospective observational design and unadjusted survival analysis. In response, we revised the title to use association-based wording and further tempered the manuscript throughout to avoid causal interpretation.

We also agree that postoperative dysphagia at POD 28 likely reflects underlying debility, frailty, and a more complicated perioperative course rather than serving as an isolated causal determinant of poorer survival. Accordingly, we revised the Discussion and Conclusion to more clearly present POD 28 dysphagia as a marker of delayed recovery and greater illness severity, while acknowledging the possibility of residual confounding.

At the same time, we believe the findings remain clinically meaningful because FOIS at POD 28 is a simple and accessible functional measure that may help identify patients with increased clinical vulnerability during the early post-transplant period. We have revised the manuscript to present the study in this more cautious and clinically grounded manner.

Title

Association of Prolonged ECMO Bridging and Perioperative Factors With Dysphagia and Survival After Lung Transplantation

Discussion

Taken together, these findings suggest that prolonged ECMO BTT may reflect both the burden of extended extracorporeal support and greater underlying illness severity, thereby identifying patients who may particularly benefit from early and systematic assessment of oropharyngeal function.(24)

However, the association between POD 28 dysphagia and post-transplant survival should be interpreted with caution, as prolonged ECMO BTT may have contributed both to the development of dysphagia and to poorer post-transplant survival.(25)

Limitation

In addition, this single-center cohort included a high proportion of patients who underwent ECMO BTT, indicating a relatively high-acuity transplant population. Because ECMO BTT itself may reflect greater underlying disease severity, it could have acted as an important confounder in the observed associations between dysphagia and clinical outcomes.

Conclusion

In this retrospective study, most LT recipients with dysphagia at POD 28 recovered swallowing function by POD 60 or by hospital discharge. Prolonged ECMO BTT and postoperative pneumonia were independently associated with dysphagia at POD 28. Dysphagia at POD 28 was also associated with poorer unadjusted 1- and 2-year survival and may serve as a clinical marker of delayed recovery and greater illness severity rather than an isolated swallowing disorder. Future prospective studies with systematic instrumental swallowing assessment and adjusted survival analyses are needed to further clarify these associations.
