# Supplementary material for: Association of prolonged ECMO bridging and perioperative factors with dysphagia and survival after lung transplantation
Source: PLoS One. 2026 Jul 9;21(7):e0353273. doi: 10.1371/journal.pone.0353273 (PMC13349096; doi:10.1371/journal.pone.0353273)
Supplement: Peer review history

# Peer review: Decision Letter 0

**DOI:** 10.1371/journal.pone.0353273.r001

**Authors:** Jamal Akhtar

15 Apr 2026

PONE-D-26-02646

Impact of Prolonged ECMO Bridging and Perioperative Factors on Dysphagia and Survival After Lung Transplantation

PLOS One

Dear Dr. Kim,

Thank you for submitting your manuscript to PLOS ONE. After careful consideration, we feel that it has merit but does not fully meet PLOS ONE’s publication criteria as it currently stands. Therefore, we invite you to submit a revised version of the manuscript that addresses the points raised during the review process.

Please submit your revised manuscript by May 30 2026 11:59PM. If you will need more time than this to complete your revisions, please reply to this message or contact the journal office at plosone@plos.org. When you're ready to submit your revision, log on to https://www.editorialmanager.com/pone/ and select the 'Submissions Needing Revision' folder to locate your manuscript file.

Please include the following items when submitting your revised manuscript:

- A letter that responds to each point raised by the academic editor and reviewer(s). You should upload this letter as a separate file labeled 'Response to Reviewers'.
- A marked-up copy of your manuscript that highlights changes made to the original version. You should upload this as a separate file labeled 'Revised Manuscript with Track Changes'.
- An unmarked version of your revised paper without tracked changes. You should upload this as a separate file labeled 'Manuscript'.

If you would like to make changes to your financial disclosure, please include your updated statement in your cover letter. Guidelines for resubmitting your figure files are available below the reviewer comments at the end of this letter.

If applicable, we recommend that you deposit your laboratory protocols in protocols.io to enhance the reproducibility of your results. Protocols.io assigns your protocol its own identifier (DOI) so that it can be cited independently in the future. For instructions see: https://journals.plos.org/plosone/s/submission-guidelines#loc-laboratory-protocols. Additionally, PLOS ONE offers an option for publishing peer-reviewed Lab Protocol articles, which describe protocols hosted on protocols.io. Read more information on sharing protocols at https://plos.org/protocols?utm_medium=editorial-email&utm_source=authorletters&utm_campaign=protocols.

As the corresponding author, your ORCID iD is verified in the submission system and will appear in the published article. PLOS supports the use of ORCID, and we encourage all coauthors to register for an ORCID iD and use it as well. Please encourage your coauthors to verify their ORCID iD within the submission system before final acceptance, as unverified ORCID iDs will not appear in the published article. *Only*  the individual author can complete the verification step; PLOS staff *cannot*  verify ORCID iDs on behalf of authors.

We look forward to receiving your revised manuscript.

Kind regards,

Jamal Akhtar

Academic Editor

PLOS One

Journal Requirements:

When submitting your revision, we need you to address these additional requirements.

1. Please ensure that your manuscript meets PLOS ONE's style requirements, including those for file naming. The PLOS ONE style templates can be found at

https://journals.plos.org/plosone/s/file?id=wjVg/PLOSOne_formatting_sample_main_body.pdf and https://journals.plos.org/plosone/s/file?id=ba62/PLOSOne_formatting_sample_title_authors_affiliations.pdf

2. Your ethics statement should only appear in the Methods section of your manuscript. If your ethics statement is written in any section besides the Methods, please delete it from any other section.

3. We note that you have indicated that there are restrictions to data sharing for this study. For studies involving human research participant data or other sensitive data, we encourage authors to share de-identified or anonymized data. However, when data cannot be publicly shared for ethical reasons, we allow authors to make their data sets available upon request. For information on unacceptable data access restrictions, please see http://journals.plos.org/plosone/s/data-availability#loc-unacceptable-data-access-restrictions.

Before we proceed with your manuscript, please address the following prompts:

a) If there are ethical or legal restrictions on sharing a de-identified data set, please explain them in detail (e.g., data contain potentially identifying or sensitive patient information, data are owned by a third-party organization, etc.) and who has imposed them (e.g., a Research Ethics Committee or Institutional Review Board, etc.). Please also provide contact information for a data access committee, ethics committee, or other institutional body to which data requests may be sent.

b) If there are no restrictions, please upload the minimal anonymized data set necessary to replicate your study findings to a stable, public repository and provide us with the relevant URLs, DOIs, or accession numbers. Please see http://www.bmj.com/content/340/bmj.c181.long for guidelines on how to de-identify and prepare clinical data for publication. For a list of recommended repositories, please see https://journals.plos.org/plosone/s/recommended-repositories. You also have the option of uploading the data as Supporting Information files, but we would recommend depositing data directly to a data repository if possible.

Please update your Data Availability statement in the submission form accordingly.

4. Please provide a complete Data Availability Statement in the submission form, ensuring you include all necessary access information or a reason for why you are unable to make your data freely accessible. If your research concerns only data provided within your submission, please write "All data are in the manuscript and/or supporting information files" as your Data Availability Statement.

5. In the online submission form, you indicated that your data is available only on request from a third party. Please note that your Data Availability Statement is currently missing the name of the contact details for the third party, such as an email address or a link to where data requests can be made. Please update your statement with the missing information.

6. When completing the data availability statement of the submission form, you indicated that you will make your data available on acceptance. We strongly recommend all authors decide on a data sharing plan before acceptance, as the process can be lengthy and hold up publication timelines. Please note that, though access restrictions are acceptable now, your entire data will need to be made freely accessible if your manuscript is accepted for publication. This policy applies to all data except where public deposition would breach compliance with the protocol approved by your research ethics board. If you are unable to adhere to our open data policy, please kindly revise your statement to explain your reasoning and we will seek the editor's input on an exemption. Please be assured that, once you have provided your new statement, the assessment of your exemption will not hold up the peer review process.

7. Thank you for stating the following financial disclosure:

This work was supported by the National Research Foundation of Korea (NRF) (No. RS-2024-00346048) and by the Korea Health Industry Development Institute (KHIDI) funded by the Ministry of Health & Welfare, Republic of Korea (RS-2024-00408722).

Please state what role the funders took in the study. If the funders had no role, please state: "The funders had no role in study design, data collection and analysis, decision to publish, or preparation of the manuscript."

If this statement is not correct you must amend it as needed.

Please include this amended Role of Funder statement in your cover letter; we will change the online submission form on your behalf.

8. If the reviewer comments include a recommendation to cite specific previously published works, please review and evaluate these publications to determine whether they are relevant and should be cited. There is no requirement to cite these works unless the editor has indicated otherwise.

[Note: HTML markup is below. Please do not edit.]

Reviewers' comments:

Reviewer's Responses to Questions

**Comments to the Author**

1. Is the manuscript technically sound, and do the data support the conclusions?

The manuscript must describe a technically sound piece of scientific research with data that supports the conclusions. Experiments must have been conducted rigorously, with appropriate controls, replication, and sample sizes. The conclusions must be drawn appropriately based on the data presented.

Reviewer #1: Yes

Reviewer #2: Partly

Reviewer #3: Partly

**********

2. Has the statistical analysis been performed appropriately and rigorously?

Reviewer #1: Yes

Reviewer #2: Yes

Reviewer #3: Yes

**********

3. Have the authors made all data underlying the findings in their manuscript fully available?

The PLOS Data policy requires authors to make all data underlying the findings described in their manuscript fully available without restriction, with rare exception (please refer to the Data Availability Statement in the manuscript PDF file). The data should be provided as part of the manuscript or its supporting information, or deposited to a public repository. For example, in addition to summary statistics, the data points behind means, medians and variance measures should be available. If there are restrictions on publicly sharing data—e.g. participant privacy or use of data from a third party—those must be specified.

Reviewer #1: No

Reviewer #2: Yes

Reviewer #3: No

**********

4. Is the manuscript presented in an intelligible fashion and written in standard English?

PLOS ONE does not copyedit accepted manuscripts, so the language in submitted articles must be clear, correct, and unambiguous. Any typographical or grammatical errors should be corrected at revision, so please note any specific errors here.

Reviewer #1: Yes

Reviewer #2: Yes

Reviewer #3: Yes

**********

5. Review Comments to the Author

Please use the space provided to explain your answers to the questions above. You may also include additional comments for the author, including concerns about dual publication, research ethics, or publication ethics. (Please upload your review as an attachment if it exceeds 20,000 characters)

Reviewer #1: Dysphagia has an important impact following lung transplantation, and I find this paper interesting and well written. I have some concerns that need to be addressed in the discussion:

1. The proportion of patients bridged with ECMO in this cohort (>50%) is substantially higher than typically reported in lung transplant populations. This raises concerns about the cohort's representativeness and limits the generalizability of the findings. Please address this in the limitations section.

2. Furthermore, the proportion of patients on prolonged ECMO is also high within the ECMO population, which is usually reserved for the sickest patients, thereby increasing the risk of confounding by indication and reducing the ability to adequately distinguish the independent effect of ECMO from underlying disease severity on the development of dysphagia. Some details on the indication for transplant (CTD-ILD, some of which have known GI involvement or idiopathic interstitial pneumonias), as well as the indication for ECMO and prolonged ECMO, will help contextualize this issue.

3. The interpretation of the association between dysphagia and post-transplant mortality is adequately discussed as more of a surrogate marker for the severity of illness than causality. Please add the prolonged ECMO confounder here, as pre-transplant ECMO, particularly when prolonged, is associated with worse 1- and 2-year survival (Rando et al. Extracorporeal membrane oxygenation as a bridge to lung transplantation: Practice patterns and patient outcomes. J Heart Lung Transplant 2024;43:77–84). In the present cohort, where ECMO use is highly prevalent, it is likely that ECMO both predisposes to the development of dysphagia and independently impacts mortality.

Reviewer #2: MAJOR CONCERNS:

1. Survival is compared between dysphagia and non-dysphagia groups using Kaplan-Meier analysis only, without multivariable Cox regression. Given that the dysphagia group had significantly more ECMO BTT, pneumonia, and longer ICU stays, the unadjusted survival difference may reflect severity of illness rather than dysphagia per se. Adjusted survival analysis is necessary to support the conclusion that dysphagia independently predicts mortality.

MINOR CONCERNS:

2. VFSS was performed in only 40 patients (32.3%), and 31 VFSS data points are missing. Reliance on FOIS alone without systematic instrumental assessment in all patients may result in underdiagnosis of silent aspiration, particularly in the non-dysphagia group. This limitation should be more prominently discussed.

STRENGTHS: Clinically important and underexplored topic; rigorous perioperative data collection; validated FOIS instrument applied at multiple time points; clear separation of ECMO BTT vs. prolonged ECMO BTT in regression models; well-written and logically structured manuscript.

Reviewer #3: In this retrospective single center cohort review, the authors provide an analysis of perioperative factors and their association with postoperative dysphagia in lung transplant recipients, as well as the survival rates in patients with postoperative dysphagia.

Chief amongst the problems with this study include the title itself, with impact implying some degree of causality. This dovetails into the principle challenge to the veracity of this paper. Dysphagia itself is likely reflective of the severity of underlying debility and/or frailty, with resultant residual confounding.

Of note, while the data may not appear to be immediately public, it appears that it should be otherwise easily obtainable from the authors and appropriately guarded for privacy reasons.

At face value, the primary takeaway from this paper appears to be that sicker or otherwise more critically ill patients with prolonged durations of such have increased rates of dysphagia with subsequently lower survival, APACHE-II scores aside. To some degree these findings seem self-evident, which may limit the effect that this publication may have.

**********

6. PLOS authors have the option to publish the peer review history of their article (what does this mean?). If published, this will include your full peer review and any attached files.

If you choose “no”, your identity will remain anonymous but your review may still be made public.

**Do you want your identity to be public for this peer review?**  For information about this choice, including consent withdrawal, please see our Privacy Policy.

Reviewer #1: No

Reviewer #2: No

Reviewer #3: **Yes:** Alexander Yuen

**********

[NOTE: If reviewer comments were submitted as an attachment file, they will be attached to this email and accessible via the submission site. Please log into your account, locate the manuscript record, and check for the action link "View Attachments". If this link does not appear, there are no attachment files.]

To ensure your figures meet our technical requirements, please review our figure guidelines: https://journals.plos.org/plosone/s/figures

You may also use PLOS’s free figure tool, NAAS, to help you prepare publication quality figures: https://journals.plos.org/plosone/s/figures#loc-tools-for-figure-preparation.

NAAS will assess whether your figures meet our technical requirements by comparing each figure against our figure specifications.

# Author comment: Author response to Decision Letter 1

**DOI:** 10.1371/journal.pone.0353273.r002

24 Apr 2026

Reviewer #1: Dysphagia has an important impact following lung transplantation, and I find this paper interesting and well written. I have some concerns that need to be addressed in the discussion:

1. The proportion of patients bridged with ECMO in this cohort (>50%) is substantially higher than typically reported in lung transplant populations. This raises concerns about the cohort's representativeness and limits the generalizability of the findings. Please address this in the limitations section.

We appreciate this important comment and agree with the reviewer. In our cohort, 69 of 124 patients (55.6%) underwent ECMO BTT, indicating that the study population reflects a relatively high-acuity transplant cohort rather than a broadly representative LT population. We have therefore revised the Limitations section to explicitly acknowledge that the high prevalence of ECMO BTT in this single-center cohort may limit the generalizability of our findings to centers with different case mix, bridging practices, or perioperative management strategies.

Limitations

In addition, this single-center cohort included a high proportion of patients who underwent ECMO BTT, indicating a relatively high-acuity transplant population. Because ECMO BTT itself may reflect greater underlying disease severity, it could have acted as an important confounder in the observed associations between dysphagia and clinical outcomes. Therefore, the generalizability of our findings to broader lung transplant populations or to centers with different bridging practices may be limited.

2. Furthermore, the proportion of patients on prolonged ECMO is also high within the ECMO population, which is usually reserved for the sickest patients, thereby increasing the risk of confounding by indication and reducing the ability to adequately distinguish the independent effect of ECMO from underlying disease severity on the development of dysphagia. Some details on the indication for transplant (CTD-ILD, some of which have known GI involvement or idiopathic interstitial pneumonias), as well as the indication for ECMO and prolonged ECMO, will help contextualize this issue.

We sincerely thank the reviewer for this important comment. We agree that prolonged ECMO support may introduce confounding by indication, as it likely reflects both ECMO-related exposure and greater underlying disease severity.

In response, we revised the Discussion to note that the Korean lung allocation system has historically prioritized medical urgency, which may partly explain the high proportion of ECMO-bridged patients in our cohort. We also agree that patients requiring prolonged ECMO BTT likely represent a particularly severe subgroup, making it difficult to fully distinguish the effect of prolonged ECMO itself from that of underlying illness severity on postoperative dysphagia.

We acknowledge that more detailed information regarding transplant indication and the indication for ECMO would further strengthen the interpretation of our findings. However, because these details were not uniformly available in a structured form across the study period, we were unable to add a reliable indication-based subgroup analysis in the current revision.

Discussion

The relatively high prevalence of ECMO BTT in our cohort should be interpreted within the context of the Korean lung allocation system, which has historically prioritized medical urgency. Under this system, candidates requiring mechanical ventilation and/or ECMO have comprised a relatively large proportion of transplant recipients in Korea, and this national allocation context may partly explain the high proportion of ECMO-bridged patients in our study.(20) Against this background, patients requiring ECMO BTT are generally recognized to have lower pretransplant functional reserve than non-BTT recipients.(21,22) In our cohort, ECMO BTT was also more common in the dysphagia group (Table 2). However, multivariable analyses demonstrated that prolonged ECMO BTT (>14 days), rather than ECMO BTT itself, was independently associated with dysphagia (Table 3). This finding is consistent with previous reports suggesting favorable outcomes when ECMO BTT is limited to ≤14 days, as well as studies showing that prolonged ECMO support, rather than ECMO use per se, increases the risk of dysphagia and aspiration in critically ill populations, including patients with COVID-19.(22,23) Taken together, these findings suggest that prolonged ECMO BTT may reflect both the burden of extended extracorporeal support and greater underlying illness severity, thereby identifying patients who may particularly benefit from early and systematic assessment of oropharyngeal function.(24)

3. The interpretation of the association between dysphagia and post-transplant mortality is adequately discussed as more of a surrogate marker for the severity of illness than causality. Please add the prolonged ECMO confounder here, as pre-transplant ECMO, particularly when prolonged, is associated with worse 1- and 2-year survival (Rando et al. Extracorporeal membrane oxygenation as a bridge to lung transplantation: Practice patterns and patient outcomes. J Heart Lung Transplant 2024;43:77–84). In the present cohort, where ECMO use is highly prevalent, it is likely that ECMO both predisposes to the development of dysphagia and independently impacts mortality.

We sincerely thank the reviewer for this important comment. We agree that prolonged ECMO BTT is an important potential confounder in interpreting the association between dysphagia and post-transplant mortality.

In response, we revised the Discussion to clarify that dysphagia at POD 28 should be viewed as a marker of greater illness severity rather than as an independent causal determinant of mortality. We also added the suggested reference by Rando et al. and noted that prolonged pre-transplant ECMO has been associated with worse post-transplant survival. Accordingly, we tempered our interpretation to acknowledge that prolonged ECMO may have contributed both to dysphagia and to poorer survival in our cohort.

Discussion

However, the association between POD 28 dysphagia and post-transplant survival should be interpreted with caution, as prolonged ECMO BTT may have contributed both to the development of dysphagia and to poorer post-transplant survival.

Reviewer 2

MAJOR CONCERNS:

1. Survival is compared between dysphagia and non-dysphagia groups using Kaplan-Meier analysis only, without multivariable Cox regression. Given that the dysphagia group had significantly more ECMO BTT, pneumonia, and longer ICU stays, the unadjusted survival difference may reflect severity of illness rather than dysphagia per se. Adjusted survival analysis is necessary to support the conclusion that dysphagia independently predicts mortality.

We sincerely thank the reviewer for this important comment. We fully agree that Kaplan–Meier analysis alone does not allow postoperative dysphagia to be interpreted as an independent predictor of mortality.

In the present study, the survival analysis was intended as a descriptive comparison of overall survival according to FOIS-defined swallowing status at POD 28, rather than as a formal adjusted prognostic model. In response to the reviewer’s comment, we revised the manuscript to avoid wording that could imply independent prediction or causality. We now describe the survival findings as an unadjusted association and interpret POD 28 dysphagia primarily as a marker of delayed recovery and greater illness severity rather than as an isolated determinant of mortality.

We also strengthened the Discussion below to acknowledge that the observed survival difference may have been influenced by important confounders, including ECMO bridge-to-transplantation, prolonged ECMO support, postoperative pneumonia, and longer ICU stay. In particular, we added that prolonged ECMO BTT may have contributed both to the development of dysphagia and to poorer post-transplant survival. We agree that adjusted time-to-event analysis would be important in future studies, and we have tempered our conclusions accordingly.

Discussion

Taken together, these findings suggest that prolonged ECMO BTT may reflect both the burden of extended extracorporeal support and greater underlying illness severity, thereby underscoring the need for early and systematic assessment of oropharyngeal function in this high-risk subgroup.(24)

However, the association between POD 28 dysphagia and post-transplant survival should be interpreted with caution, as prolonged ECMO BTT may have contributed both to the development of dysphagia and to poorer post-transplant survival.

Limitation

In addition, this single-center cohort included a high proportion of patients who underwent ECMO BTT, indicating a relatively high-acuity transplant population. Because ECMO BTT itself may reflect greater underlying disease severity, it could have acted as an important confounder in the observed associations between dysphagia and clinical outcomes. Therefore, the generalizability of our findings to broader lung transplant populations or to centers with different bridging practices may be limited.

MINOR CONCERNS:

2. VFSS was performed in only 40 patients (32.3%), and 31 VFSS data points are missing. Reliance on FOIS alone without systematic instrumental assessment in all patients may result in underdiagnosis of silent aspiration, particularly in the non-dysphagia group. This limitation should be more prominently discussed.

We thank the reviewer for this important point. We apologize for the ambiguity in the table. The 31 missing values refer to APACHE-II scores, not VFSS. VFSS was performed selectively in patients with clinical suspicion of dysphagia rather than systematically in all patients. We agree, however, that reliance on FOIS without universal instrumental swallowing assessment may have led to underrecognition of silent aspiration, particularly in patients classified as non-dysphagic based on FOIS alone. We have clarified this point in the revised manuscript and emphasized it more explicitly in the Limitations section.

Limitation

Second, FOIS does not fully characterize swallowing physiology, as it cannot detect silent aspiration or penetration. Although FOIS is readily applicable in routine clinical practice, it lacks the objective visualization provided by VFSS or FEES, and instrumental swallowing assessments were performed in only a subset of patients in this study.

Reviewer #3: In this retrospective single center cohort review, the authors provide an analysis of perioperative factors and their association with postoperative dysphagia in lung transplant recipients, as well as the survival rates in patients with postoperative dysphagia.

Chief amongst the problems with this study include the title itself, with impact implying some degree of causality. This dovetails into the principle challenge to the veracity of this paper. Dysphagia itself is likely reflective of the severity of underlying debility and/or frailty, with resultant residual confounding.

Of note, while the data may not appear to be immediately public, it appears that it should be otherwise easily obtainable from the authors and appropriately guarded for privacy reasons.

At face value, the primary takeaway from this paper appears to be that sicker or otherwise more critically ill patients with prolonged durations of such have increased rates of dysphagia with subsequently lower survival, APACHE-II scores aside. To some degree these findings seem self-evident, which may limit the effect that this publication may have.

We sincerely thank the reviewer for this thoughtful and careful assessment. We agree that the term “impact” in the original title may imply a degree of causality that is not fully supported by our retrospective observational design and unadjusted survival analysis. In response, we revised the title to use association-based wording and further tempered the manuscript throughout to avoid causal interpretation.

We also agree that postoperative dysphagia at POD 28 likely reflects underlying debility, frailty, and a more complicated perioperative course rather than serving as an isolated causal determinant of poorer survival. Accordingly, we revised the Discussion and Conclusion to more clearly present POD 28 dysphagia as a marker of delayed recovery and greater illness severity, while acknowledging the possibility of residual confounding.

At the same time, we believe the findings remain clinically meaningful because FOIS at POD 28 is a simple and accessible functional measure that may help identify patients with increased clinical vulnerability during the early post-transplant period. We have revised the manuscript to present the study in this more cautious and clinically grounded manner.

Title

Association of Prolonged ECMO Bridging and Perioperative Factors With Dysphagia and Survival After Lung Transplantation

Discussion

Taken together, these findings suggest that prolonged ECMO BTT may reflect both the burden of extended extracorporeal support and greater underlying illness severity, thereby identifying patients who may particularly benefit from early and systematic assessment of oropharyngeal function.(24)

However, the association between POD 28 dysphagia and post-transplant survival should be interpreted with caution, as prolonged ECMO BTT may have contributed both to the development of dysphagia and to poorer post-transplant survival.(25)

Limitation

In addition, this single-center cohort included a high proportion of patients who underwent ECMO BTT, indicating a relatively high-acuity transplant population. Because ECMO BTT itself may reflect greater underlying disease severity, it could have acted as an important confounder in the observed associations between dysphagia and clinical outcomes.

Conclusion

In this retrospective study, most LT recipients with dysphagia at POD 28 recovered swallowing function by POD 60 or by hospital discharge. Prolonged ECMO BTT and postoperative pneumonia were independently associated with dysphagia at POD 28. Dysphagia at POD 28 was also associated with poorer unadjusted 1- and 2-year survival and may serve as a clinical marker of delayed recovery and greater illness severity rather than an isolated swallowing disorder. Future prospective studies with systematic instrumental swallowing assessment and adjusted survival analyses are needed to further clarify these associations.

Attachment Submitted filename: Response letter.docx

# Peer review: Decision Letter 1

**DOI:** 10.1371/journal.pone.0353273.r003

**Authors:** Jamal Akhtar, Jamal Akhtar

22 Jun 2026

Association of Prolonged ECMO Bridging and Perioperative Factors With Dysphagia and Survival After Lung Transplantation

PONE-D-26-02646R1

Dear Dr. Kim,

We’re pleased to inform you that your manuscript has been judged scientifically suitable for publication and will be formally accepted for publication once it meets all outstanding technical requirements.

Within one week, you’ll receive an e-mail detailing the required amendments. When these have been addressed, you’ll receive a formal acceptance letter and your manuscript will be scheduled for publication.

An invoice will be generated when your article is formally accepted. Please note, if your institution has a publishing partnership with PLOS and your article meets the relevant criteria, all or part of your publication costs will be covered. Please make sure your user information is up-to-date by logging into Editorial Manager at Editorial Manager® and clicking the ‘Update My Information' link at the top of the page. For questions related to billing, please contact billing support.

If your institution or institutions have a press office, please notify them about your upcoming paper to help maximize its impact. If they’ll be preparing press materials, please inform our press team as soon as possible -- no later than 48 hours after receiving the formal acceptance. Your manuscript will remain under strict press embargo until 2 pm Eastern Time on the date of publication. For more information, please contact onepress@plos.org.

Kind regards,

Jamal Akhtar

Academic Editor

PLOS One

Additional Editor Comments (optional):

Reviewers' comments:

Reviewer's Responses to Questions

**Comments to the Author**

1. If the authors have adequately addressed your comments raised in a previous round of review and you feel that this manuscript is now acceptable for publication, you may indicate that here to bypass the “Comments to the Author” section, enter your conflict of interest statement in the “Confidential to Editor” section, and submit your "Accept" recommendation.

Reviewer #2: All comments have been addressed

Reviewer #3: All comments have been addressed

**********

2. Is the manuscript technically sound, and do the data support the conclusions?

The manuscript must describe a technically sound piece of scientific research with data that supports the conclusions. Experiments must have been conducted rigorously, with appropriate controls, replication, and sample sizes. The conclusions must be drawn appropriately based on the data presented.

Reviewer #2: Yes

Reviewer #3: Yes

**********

3. Has the statistical analysis been performed appropriately and rigorously?

Reviewer #2: Yes

Reviewer #3: Yes

**********

4. Have the authors made all data underlying the findings in their manuscript fully available?

The PLOS Data policy requires authors to make all data underlying the findings described in their manuscript fully available without restriction, with rare exception (please refer to the Data Availability Statement in the manuscript PDF file). The data should be provided as part of the manuscript or its supporting information, or deposited to a public repository. For example, in addition to summary statistics, the data points behind means, medians and variance measures should be available. If there are restrictions on publicly sharing data—e.g. participant privacy or use of data from a third party—those must be specified.

Reviewer #2: Yes

Reviewer #3: Yes

**********

5. Is the manuscript presented in an intelligible fashion and written in standard English?

PLOS ONE does not copyedit accepted manuscripts, so the language in submitted articles must be clear, correct, and unambiguous. Any typographical or grammatical errors should be corrected at revision, so please note any specific errors here.

Reviewer #2: Yes

Reviewer #3: Yes

**********

6. Review Comments to the Author

Please use the space provided to explain your answers to the questions above. You may also include additional comments for the author, including concerns about dual publication, research ethics, or publication ethics. (Please upload your review as an attachment if it exceeds 20,000 characters)

Reviewer #2: All comments have been addressed and I appreciate the detailed and thoughtful responses provided by the authors

Reviewer #3: I believe that my comments have been addressed. The nature of the study remains self-evident, but there is suggestion that dysphagia as assessed by FOIS at POD 28 can be utilized as a functional measure to assist with identifying patients at higher risk of postoperative complications, as a reflection of overall frailty.

**********

7. PLOS authors have the option to publish the peer review history of their article (what does this mean?). If published, this will include your full peer review and any attached files.

If you choose “no”, your identity will remain anonymous but your review may still be made public.

**Do you want your identity to be public for this peer review?**  For information about this choice, including consent withdrawal, please see our Privacy Policy.

Reviewer #2: No

Reviewer #3: **Yes:** Alexander Yuen

**********

# Editor report: Acceptance letter

**DOI:** 10.1371/journal.pone.0353273.r004

**Authors:** Jamal Akhtar, Jamal Akhtar

PONE-D-26-02646R1

PLOS One

Dear Dr. Kim,

I'm pleased to inform you that your manuscript has been deemed suitable for publication in PLOS One. Congratulations! Your manuscript is now being handed over to our production team.

At this stage, our production department will prepare your paper for publication. This includes ensuring the following:

* All references, tables, and figures are properly cited

* All relevant supporting information is included in the manuscript submission,

* There are no issues that prevent the paper from being properly typeset

You will receive further instructions from the production team, including instructions on how to review your proof when it is ready. Please keep in mind that we are working through a large volume of accepted articles, so please give us a few days to review your paper and let you know the next and final steps.

Lastly, if your institution or institutions have a press office, please let them know about your upcoming paper now to help maximize its impact. If they'll be preparing press materials, please inform our press team within the next 48 hours. Your manuscript will remain under strict press embargo until 2 pm Eastern Time on the date of publication. For more information, please contact onepress@plos.org.

You will receive an invoice from PLOS for your publication fee after your manuscript has reached the completed accept phase. If you receive an email requesting payment before acceptance or for any other service, this may be a phishing scheme. Learn how to identify phishing emails and protect your accounts at https://explore.plos.org/phishing.

If we can help with anything else, please email us at customercare@plos.org.

Thank you for submitting your work to PLOS ONE and supporting open access.

Kind regards,

PLOS ONE Editorial Office Staff

on behalf of

Dr. Jamal Akhtar

Academic Editor

PLOS One
